# Central nervous system relapse in patients with breast cancer is associated with advanced stages, with the presence of circulating occult tumor cells and with the HER2/neu status

**DOI:** 10.1186/bcr1516

**Published:** 2006-07-17

**Authors:** John Souglakos, Lambros Vamvakas, Stella Apostolaki, Maria Perraki, Zacharenia Saridaki, Irine Kazakou, Athanasios Pallis, Charalambos Kouroussis, Nikos Androulakis, Kostas Kalbakis, Georgia Millaki, Dimitris Mavroudis, Vassilis Georgoulias

**Affiliations:** 1 Department of Medical Oncology, University General Hospital of Heraklion, Crete, Greece; 2 Laboratory of Tumor Cell Biology, School of Medicine, University of Crete, Heraklion, Crete, Greece

## Abstract

**Introduction:**

To evaluate the incidence of central nervous system (CNS) involvement in patients with breast cancer treated with a taxane-based chemotherapy regimen and to determine predictive factors for CNS relapse.

**Methods:**

The medical files of patients with early breast cancer (*n *= 253) or advanced stage breast cancer (*n *= 239) as well of those with other solid tumors (*n *= 336) treated with or without a taxane-based chemotherapy regimen during a 42-month period were reviewed. HER2/neu overexpression was identified by immunohistochemistry, whereas cytokeratin 19 (CK-19) mRNA-positive circulating tumor cells (CTCs) in the peripheral blood were identified by real-time PCR.

**Results:**

The incidence of CNS relapse was similar in patients suffering from breast cancer or other solid tumors (10.4% and 11.4%, respectively; *P *= 0.517). The incidence of CNS relapse was significantly higher in breast cancer patients with advanced disease (*P *= 0.041), visceral disease and bone disease (*P *= 0.036), in those who were treated with a taxane-containing regimen (*P *= 0.024), in those with HER2/neu-overexpressing tumors (*P *= 0.022) and, finally, in those with detectable CK-19 mRNA-positive CTCs (*P *= 0.008). Multivariate analysis revealed that the stage of disease (odds ratio, 0.23; 95% confidence interval, 0.007–0.23; *P *= 0.0001), the HER2/neu status (odds ratio, 29.4; 95% confidence interval, 7.51–101.21; *P *= 0.0001) and the presence of CK-19 mRNA-positive CTCs (odds ratio, 8.31; 95% confidence interval, 3.97–12.84; *P *= 0.001) were independent predictive factors for CNS relapse.

**Conclusion:**

CNS relapses are common among breast cancer patients treated with a taxane-based chemotherapy regimen, patients with HER2/neu-positive tumor and patients with CK-19 mRNA-positive CTCs.

## Introduction

Breast cancer is the second leading cause of brain metastases [1,2]. Based on autopsy reports, the incidence of central nervous system (CNS) metastases among patients with breast cancer is as high as 23% [3] but only 10–15% of these patients will develop clinically overt (CNS) manifestations [2]. The problem of brain metastases will probably become more important since patients survive longer [3,4], as a consequence of more accurate diagnostic tools, of more aggressive and effective adjuvant and locoregional treatment but, also, as a consequence of better palliative therapy.

The factors influencing the development of brain metastases in patients with breast cancer are poorly understood. Previous studies have reported that negative hormonal receptor status [5,6], HER2/neu overexpression [7,8] and the presence of lung metastases [9,10] were associated with a higher risk for the development of brain metastases. In addition, two other studies reported a high incidence of CNS metastatic disease in patients with locally advanced and metastatic breast cancer treated with a taxane-containing chemotherapy regimen [9-11].

The reasons for the association between treatment of breast cancer with a taxane-containing chemotherapy regimen and an increased incidence of CNS involvement are not clear. Although taxanes are very lipophilic, their concentration in the CNS is very low after their intravenous administration [12,13]. Moreover, preclinical data suggest that taxanes are unable to penetrate the intact blood–brain barrier; in these models, the concentration of radiolabeled paclitaxel in the cerebrospinal fluid was found to be significantly lower than in other organs, and thus was undetectable in the brain, in the spinal cord or in any other site of the CNS [14].

It has more recently been proposed that paclitaxel is exported from the *p*-glycoprotein and other ATP-binding cassette transporters placed at the luminal membrane of brain capillaries, as an explanation for the low concentrations of taxanes in the CNS [15].

Furthermore, the detection of cytokeratin 19 (CK-19) and of mRNA-positive circulating tumor cells (CTCs) in the peripheral blood and the bone marrow of patients with breast cancer was correlated with increased incidence of relapse in several studies [16,17]. The possible correlation of this finding with an increased incidence of CNS relapse was not reported in any of these studies.

During the past years we have frequently observed patients with breast cancer treated with a taxane-containing chemotherapy regimen, either in the adjuvant setting or in the metastatic setting, presenting CNS involvement as the only evidence of disease progression. We were therefore interested to evaluate the incidence of CNS metastases in patients with early and advanced breast cancer treated with a taxane-containing chemotherapy regimen and to identify predictive factors for CNS relapse.

## Materials and methods

The medical files of all patients submitted to the Department of Medical Oncology of the University General Hospital of Heraklion, Crete during the period January 2000–June 2003 were retrospectively reviewed for the development of CNS metastatic disease. Patients with a CNS relapse as the first and only indication of metastatic disease or patients with isolated CNS involvement diagnosed during or within three months of the completion of previous chemotherapy were considered. Four groups of patients with CNS metastatic disease were identified: group A, patients with breast cancer treated with a taxane-containing regimen; group B, patients with breast cancer treated with a nontaxane-containing regimen; group C, patients with another type of solid tumor type treated with a taxane-containing regimen; and group D, patients with another type of solid tumor treated with a nontaxane-containing regimen. Groups B–D were used as controls.

For each patient with CNS metastatic disease, information concerning tumor characteristics (histological type, grade, HER2/neu status by immunohistochemistry with the CB-11 antibody, and estrogen receptor and progesterone receptor status by immunohistochemistry), prior treatment (response to treatment, time to tumor progression), type of CNS involvement (cerebral metastasis, leptomeningeal carcinomatosis), treatment of CNS metastasis and overall survival were recorded. Leptomeningeal metastasis ought to have been confirmed by CNS cytology. All medical files were reviewed by two investigators (JS and LV). Computerized tomography of the chest and abdomen and a bone scan were performed at the time of CNS relapse as usual practice in our center. All patients signed informed consent for the participation in clinical trials and the use of the biologic material. The study was approved by the Ethics and Scientific Committees of our Institution.

Peripheral blood mononuclear cells were prospectively obtained from patients with either early breast cancer (stage I and stage II) or advanced breast cancer (stage III and stage IV) before the initiation of any chemotherapy, in order to identify CTCs that express the CK-19 mRNA-positive cells. A quantitative real-time RT-PCR assay was used for the detection of CK-19 mRNA-positive CTCs, as previously described [16,17]. We evaluated the presence of CK-19 mRNA-positive CTCs in association with CNS relapse.

Statistical analysis was performed using the chi-square test for the analysis of the two-dimensional contingency tables. The two-sided significance level was set at *P *< 0.05. A linear regression analysis was performed to identify independent predictive factors significantly influencing the incidence of CNS involvement. Kaplan–Meier analysis was used in order to estimate the probability of survival [18].

## Results

### Patient's characteristics

One thousand and forty-five previously untreated patients with histologically or cytologically confirmed solid tumors were enrolled in the present analysis of CNS involvement during the predetermined 42-month study period. There were 305 patients in group A, 187 patients in group B, 416 patients in group C and 137 patients in group D. Two hundred and fifty-three patients and 239 patients presented early breast cancer (stage I and stage II) and locally advanced/metastatic breast cancer (stage III and stage IV), respectively. There was no difference in terms of median age, menopausal status and hormone receptor or HER2/neu status in patients with early and advanced breast cancer (Table 1). Taxane-containing chemotherapy regimens were administered either in the adjuvant setting or as frontline chemotherapy for metastatic disease in 50.2% and 74.5% of patients, respectively (Table 1).

### CNS relapse in breast cancer patients

Fifty-one patients (10.4%) and 61 patients (11.4%) with breast cancer or other solid tumors developed CNS metastatic disease during the study period (*P *= 0.517).

The CNS involvement in breast cancer patients was cerebral metastasis in 44 patients (86.3%), leptomeningeal involvement in five patients (9.8%) and both cerebral metastasis and leptomeningeal involvement in two patients (3.9%). Two leptomeningeal metastases were recorded in patients with lobular tumor, and five in patients with ductal tumors.

In patients with other solid tumors, CNS involvement was cerebral metastasis in 49 patients (80.3%), leptomeningeal disease in nine patients (14.8%) and both cerebral metastasis and leptomeningeal disease in three patients (4.9%).

The median progression-free interval for the whole group of patients with breast cancer who developed CNS relapse was 5 months (range, 1–9 months), and that for patients with other solid tumors was 5.5 months (range, 1–7.5 months). The median progression-free intervals were 7 months (range, 4–9 months) and 4.5 months (range, 1–6 months) in patients with early breast cancer and advanced/metastatic breast cancer, respectively.

Forty-two of the 51 relapsed patients (82.4%) were treated with whole brain radiotherapy, four patients (7.8%) with whole brain and spinal cord radiotherapy, and one patient with intrathecal chemotherapy and radiotherapy, while four patients (4.7%) did not receive further treatment. The median survival time for the whole group of CNS relapsed breast cancer patients was 8.5 months (range, 1–25 months) and the 1-year survival rate was 12.3%. The median survival times were 10 months (range, 2–25 months) and 8 months (range, 1–19 months) for patients with early breast cancer and advanced/metastatic breast cancer, respectively.

### CNS involvement according to the biological and clinical characteristics of breast cancer

Table 1 indicates that the incidence of CNS involvement was higher in premenopausal patients than in postmenopausal patients (60.8% versus 39.2%, respectively; *P *= 0.071). In addition, CNS relapses were equally distributed in patients with estrogen receptor-positive and estrogen receptor-negative tumors (*P *= 0.51). Conversely, a significantly higher incidence of CNS relapses was observed in patients with advanced/metastatic disease compared with early stage disease (41 patients (17.1%) and 10 patients (3.9%), respectively; *P *= 0.041), in patients with visceral (*n *= 27 patients, 52.9%) disease compared with only soft tissue metastases (*n *= 6, 11.8%; *P *= 0.036), in patients with HER2/neu-overexpressing tumors compared with HER2/neu-negative tumors (52.9% and 31.4%, respectively; *P *= 0.002), in patients with detectable occult CK-19 mRNA-positive CTCs in the peripheral blood compared with those without CK-19 mRNA-positive cells (64.7% and 35.3%, respectively; *P *= 0.041), and in patients who received a taxane-containing chemotherapy regimen (group A) compared with those who were treated with a nontaxane-containing chemotherapy regimen (group B) (76.5% and 23.5%, respectively; *P *= 0.024).

In addition, 43 patients with metastatic disease and HER-2 overexpression in the primary tumor were treated with Trastuzumab. Sixteen (37.2%) of those patients presented CNS relapse during their treatment. Table 2 indicates that in patients with other solid tumors the used chemotherapy regimen did not influence the incidence of CNS involvement (group C, 7.6%; group D, 6.5%; *P *= 0.196).

### CNS relapse as the unique manifestation of disease progression

In 40 out of 51 patients (78.4%) the CNS relapse was the unique manifestation of disease progression. Thirty-two (80%) and eight (20%) of these relapses occurred in group A patients and group B patients, respectively (*P *= 0.067). Table 3 indicates that the incidence of CNS involvement as the unique site of disease progression was higher in stage III/stage IV group A patients (65%) than in group B patients (12.5%; *P *= 0.082) as well as in stage I/stage II patients (15% and 7.5%, respectively; *P *= 0.114). In addition, among the 40 patients with early breast cancer and advanced/metastatic breast cancer who presented an isolated CNS relapse, 23 patients (57.5%) had HER2/neu-positive (grade 2+ and grade 3+) tumors and 11 patients (27.5%) had HER2/neu-negative (grade 0+ and grade 1+) tumors (*P *= 0.002). Similarly, 26 of these 40 patients (65%) displayed detectable CK-19 mRNA-positive CTCs in the peripheral blood and 14 patients (35%) did not (*P *= 0.004).

### Multivariate analysis

Table 5 demonstrates that the stage of disease (odds ratio, 0.23; *P *< 0.0001), the detection of CK-19 mRNA-positive CTCs (odds ratio, 8.31; *P *= 0.001) and the HER2/neu status (odds ratio, 29.41; *P *< 0.0001) emerged as independent predictive factors for CNS relapse. A trend towards a significant difference was also observed for the response to treatment (*P *= 0.096; Table 5). Similarly, the multivariate analysis of the different parameters performed in the 40 patients in whom CNS relapse was the only manifestation of disease progression revealed the same predictive factors for CNS relapse (stage of the disease: odds ratio, 0.28 *P *< 0.001; detection of CK-19 mRNA-positive CTCs: odds ratio, 8.97; *P *< 0.001; HER2/neu status: odds ratio, 30.42; *P *< 0.0001). A trend towards a significant difference for the response to treatment was again detected (*P *= 0.097).

## Discussion

The development of brain metastases is usually a late event in the natural history of uncontrolled cancer. Despite increased interest during past years there are very few established predictive factors associated with the occurrence of CNS metastatic disease in patients with breast cancer. It is a common clinical experience that brain metastases occur more frequently in young women with large and/or aggressive tumors [4]. Several studies have also demonstrated that the negative hormonal receptor status and the presence of lung metastases may be associated with a higher risk of developing brain metastases [5,6,8,10]. In addition, recent studies reported that breast cancer patients who received a taxane-containing chemotherapy regimen had a significantly higher incidence of CNS metastases compared with that of patients treated with a nontaxane-containing regimen [9,11]. There are also data indicating an increased risk for brain metastases in breast cancer patients receiving trastuzumab [8,19-22].

The results of the present study demonstrate that the overall incidence of CNS metastatic disease in patients with breast cancer (both early breast cancer and advanced/metastatic breast cancer) is not different from that observed in patients with other types of solid tumors. The multivariate analysis, however, demonstrated that the stage of the disease, the HER2/neu status (grade 2+ or grade 3+ by immunohistochemistry) and the presence of CK-19 mRNA-positive CTCs represent independent predictive factors for CNS relapse. The same parameters emerged as independent predictive factors in the subgroup of breast cancer patients in whom the CNS involvement was the unique site of disease progression.

The predictive value of the HER2/neu status already reported [8,9] was further confirmed in the present study. Moreover, it has been shown that the HER2/neu status of the brain metastases is similar and highly concordant (97%) with that of the primary tumor, suggesting that the HER2/neu status of the primary tumor is predictive of the HER2/neu status of the tumors. The time to develop CNS metastatic disease seems to be shorter in patients with HER2/neu-positive brain lesions, demonstrating the importance of evaluating the HER2/neu status early in the diagnosis of breast cancer in order to provide information on the patient's prognosis and to assist clinical decision-making. It has been reported that 25% of metastatic breast cancer patients developed CNS metastatic disease during treatment with trastuzumab [20], and that nearly 10% of patients receiving trastuzumab in combination with chemotherapy developed isolated CNS metastases as the first site of tumor progression [22]. In addition, almost 15% of patients with disseminated breast cancer had occult CNS metastases and the only predictive factors for this evolution were the number of sites involved and the HER2/neu status [8].

A possible explanation for these observations could be a predilection of HER2/neu tumors to developed CNS metastases. Alternatively, we cannot exclude that trastuzumab may control the systemic disease, leading to an improved survival [23]. Since trastuzumab cannot cross the blood–brain barrier, however, the CNS remains a vulnerable 'sanctuary site'. Irrespective of the reasons for the observed increased incidence of brain metastases in patients with HER2/neu-overexpressing breast cancer, the practical problem remains the development of CNS disease, which has an extremely poor prognosis. It is therefore important to develop a risk assessment and therapeutic strategy in order to prevent and/or control CNS disease, aiming at prolonging patient survival.

We also observed that the incidence of brain metastases was significantly higher in patients with visceral disease than soft tissue disease, despite the fact that this parameter did not emerge as an independent predictive factor in the multivariate analysis. Although we did not discriminate between pulmonary and other visceral localizations of disease, this finding is in agreement with other studies [8-10] reporting a higher incidence of brain metastases in patients with lung metastases (nearly 30%). This higher incidence of brain metastases in patients with visceral disease is common evidence in retrospective studies [9,20] and should probably be attributed to the more aggressive biologic behavior of these tumors and the higher probability of hematogeneous dissemination of malignant cells in these patients. We have also reported that patients with CK-19 mRNA-positive CTCs have increased incidence for relapse with metastatic disease [17,24]. Moreover, our observation that the detection of CK-19 mRNA-positive CTCs was associated with an increased risk of CNS involvement in patients with breast cancer further support the hypothesis.

This finding was not surprising since the detection of CK-19 mRNA-positive tumor cells in the peripheral blood [17,24] or in the bone marrow [25,26] represents an independent predictive and prognostic factor for early clinical relapse and death from the disease. Indeed, the circulating CK-19 mRNA-positive tumor cells have the possibility, through the blood vasculature, to reach every tissue. The development of clinically overt metastatic disease is not a random phenomenon according to the 'seed and soil' theory, since the homing ability of these circulating tumor cells is determined by their biological characteristics as well as by other biological factors of the local environment [27]. Maguire and colleagues have reported the detection of cytokeratin-positive cells in bone marrow aspirates in 12 out of 12 cancer patients with CNS relapse; in nine of these patients, bone marrow occult cells were the only evidence for systemic spread [28]. The validation of the predictive value of CK-19 mRNA-positive cells for CNS relapse, however, requires a prospective study with a higher number of patients, a more prolonged follow-up period and further molecular and biological characterization of peripheral blood CK-19 mRNA-positive occult tumor cells.

In the present study it was also possible to confirm the initial clinical observation that breast cancer patients who receive a taxane-containing chemotherapy regimen have a significantly higher incidence of CNS metastases compared with that of patients treated with a nontaxane-containing regimen. Nevertheless, this parameter did not emerge as an independent predictive factor in the multivariate analysis. It is important to note, however, that a similar difference was not observed in patients with other types of solid tumor treated or not with a taxane-containing chemotherapy regimen.

Taxanes are highly effective in breast cancer and they prolong the survival of patients with advanced disease. It is also well known that CNS involvement is more frequent in the late stages of the disease. A possible explanation could therefore be that the increase in survival and the prolonged period of advanced disease is correlated with the increased frequency of CNS relapse. It is important to note that such a correlation could represent a confounding factor, in a retrospective analysis. For example, someone can hypothesize that physicians may have chosen more aggressive treatment for patients with aggressive tumors. These patients also showed increased incidence for CNS relapse. Although this observation could be explained with the 'sanctuary site' hypothesis, experimental studies in mice have demonstrated that the blood–brain barrier is intact inside and around brain metastases smaller than 0.2 mm^2 ^but not in larger lesions [29]. This finding implies that the barrier should not be a major obstacle to using chemotherapy in the treatment of brain metastases, at least in the advanced stages of the disease. In addition, more recent studies have shown that paclitaxel is exported by the *p*-glycoprotein and other ATP-binding cassette transporters placed at the luminal membrane of brain capillaries [15].

The aforementioned data suggest that taxanes may not penetrate well into the CNS, and therefore the CNS may represent tumor 'sanctuary' sites for taxane-containing chemotherapy regimens. A difference in the incidence of CNS relapses between patients with breast cancer and other solid tumors treated with taxanes was observed. This difference in the incidence of CNS relapse between patients with breast cancer and other solid tumors could be explained by the higher efficacy of the taxanes in breast cancer which is associated with increased survival in comparison with other solid tumors.

We have previously reported that the use of Transtuzumab could decrease or eradicate CK-19 mRNA-positive CTCs [30]. Based on these results we have initiated a randomized clinical trial to evaluate the effect of Transtuzumab administration on the disease-free survival of patients with early breast cancer and CK-19 and HER-2 mRNA-positive CTCs. The incidence of CNS relapse in correlation with the detection of CK-19 mRNA-positive CTCs will be prospectively evaluated in this ongoing study.

## Conclusion

Our study raises some interesting approaches that could be of relevance in the daily clinical practice: evaluating the incorporation of prophylactic CNS irradiation in patients with breast cancer who present unfavorable predictive factors for CNS relapse (that is, HER2/neu tumors, visceral metastases with detectable CK-19 mRNA-positive CTCs); the evaluation and development of new therapeutic approaches and strategies to treat occult tumor cells before the development of overt clinical relapse; and, since in the future we are going to treat more patients with isolated brain metastases, the need to develop new and more aggressive diagnostic, therapeutic and follow-up approaches. Further studies are therefore warranted to better understand and investigate the characteristics of this particular clinical evolution of patients with breast cancer and, thus, to more effectively treat these patients.

## Abbreviations

CK-19 = cytokeratin 19; CNS = central nervous system; CTCs = circulating tumor cells; PCR = polymerase chain reaction; RT = reverse transcriptase.

## Competing interests

The authors declare that they have no competing interests.

## Authors' contributions

JS made substantial contributions to the conception and design of the study, the acquisition and interpretation of data, and was involved in drafting the manuscript. LV made substantial contributions to the acquisition and interpretation of the data. SA and MP carried out the molecular studies. ZS made substantial contributions to the acquisition and interpretation of the data. IK made substantial contributions to data acquisition. AP participated in the design of the study and performed the statistical analysis. ChK made substantial contributions to the acquisition and interpretation of the data. NA made substantial contributions to data interpretation and was involved in drafting the manuscript. KK made substantial contributions to the acquisition of data. GM carried out the molecular studies. DM and VG conceived of the study, participated in its design and coordination, and helped to draft the manuscript. All authors read and approved the final manuscript.

## Figures and Tables

**Table 1:** Breast cancer patient characteristics during the period January 2000–June 2003

	Early stage breast cancer (*n *= 253)	Advanced/metastatic stage breast cancer (*n *= 239)	Central nervous system relapse (*n *= 51)	*P *value
Age (years) (median (range))	51 (28–75)	53 (31–81)	55 (30–70)	
Menopausal status				
Premenopausal	109 (43.1)	108 (45.2)	31 (60.8)	0.071
Postmenopausal	144 (56.9)	131 (54.8)	20 (39.2)	
Estrogen receptor status				
Positive	118 (46.6)	126 (52.7)	25 (49)	0.51
Negative	120 (47.4)	105 (43.9)	25 (49)	
Unknown	15 (6)	8 (3.3)	1 (2)	
Histology				
Ductal	195 (77)	182 (76.1)	38 (74.5)	0.287^b^
Lobular	20 (7.9)	21 (8.7)	5 (9.8)	
Other	38 (15.1)	36 (16.2)	8 (15.6)	
HER-2 status				
Positive	31 (12.3)	43 (18)	27 (52.9)	0.002
Negative	130 (51.4)	124 (51.9)	16 (6.2)	
Unknown	92 (36.4)	72 (30.1)	8 (4.8)	
Dominant disease localization at baseline				
Viscera	-	134 (56)	27 (66)^α^	0.036^c^
Soft tissues	-	63 (26.3)	6 (20)	
Bones	-	42 (17.5)	8 (14)	
Cytokeratin 19 mRNA-positive cells				
Positive	29 (12)	49 (21)	33 (65)	0.041
Negative	224 (88)	190 (79)	18 (35)	
Taxane-based treatment				
Yes	127	178 (75)	39 (12.8)	
No	126	61 (25)	12 (6.4)	
Response to chemotherapy	-			
Complete response + partial response	-	117 (49)	23 (56.3)^a^	0.59
Stable disease	-	69 (29)	8 (18.7)	0.42
Progressive disease	-	53 (22)	10 (25)	0.55

Data presented as *n *(%). ^a^Proportion of patients corresponds to the 41 patients with advanced/metastatic disease. ^*b*^*P *value corresponds to the incidence of central nervous system relapses between patients with lobular and ductal histology. ^*c*^*P *value corresponds to the incidence of central nervous system relapses between patients with visceral disease localization and other disease sites (soft tissue + bones).

**Table 2:** Incidence of central nervous system (CNS) relapses in patients with breast cancer and types other of solid tumor according to the administered chemotherapy regimen

Patients	CNS relapses		*P *value
		
	*n*	Percentage	
Group A (*n *= 305)	39	12.8	0.024^a^
Group B (*n *= 187)	12	6.5	
Group C (*n *= 416)	32	7.6	0.196^b^
Group D (*n *= 137)	9	6.5	

Group A, patients with breast cancer treated with a taxane-containing regimen; group B, patients with breast cancer treated with a nontaxane-containing regimen; group C, patients with another type of solid tumor type treated with a taxane-containing regimen; and group D, patients with another type of solid tumor treated with a nontaxane-containing regimen. ^*a*^*P *value corresponds to the incidence of CNS relapses between groups A and B. ^*b*^*P *value corresponds to the incidence of CNS relapses between groups C and D.

**Table 3:** Incidence of central nervous system metastasis as the only manifestation of disease progression according to the stage of disease

	CNS relapses (*n *= 40)		*P *value
		
	Group A (*n *= 32)	Group B (*n *= 8)	
Stage I/stage II	6 (15%)	3 (7.5%)	0.367
Stage IIIb/stage IV	26 (65%)	5 (12.5%)	0.143
All stages	32 (80%)	8 (20%)	0.048

Group A, patients with breast cancer treated with a taxane-containing regimen; group B, patients with breast cancer treated with a nontaxane-containing regimen.

**Table 4:** Incidence of central nervous system relapses as the only manifestation of disease progression in breast cancer patients according to the detection of cytokeratin 19 mRNA-positive cells in peripheral blood

Cytokeratin 19 mRNA cells	CNS relapses (*n *= 40)		*P *value
		
	*n*	Percentage	
Positive (*n *= 78)	26	33.3	0.004
Negative (*n *= 414)	14	3.4	

**Table 5:** Multivariate analysis of risk factors for central nervous system metastasis for the whole study population

Variable	Odds ratio	95% confidence interval	*P *value
Stage of disease	0.23	0.007–0.34	0.0001
Taxane-based regimen	1.61	0.71–3.63	0.246
Line of therapy	1.64	0.67–2.76	0.208
Cytokeratin 19 mRNA-positive circulating tumor cells	8.31	3.97–12.84	0.001
Premenopausal	1.9	0.58–6.45	0.278
Response to treatment	0.81	0.57–4.65	0.096
HER-2 status	29.41	7.51–101.21	0.0001
Visceral disease	1.8	0.84–3.17	0.211

Dependent variable, central nervous system relapses.
